# Health Tract No. 27

**Published:** 1862-01

**Authors:** 


### HEALTH TRACT NO. 27.

### VALUABLE KNOWLEDGE.

Bites and Stings of insects and snakes have been cured by instantly washing the parts freely with spirits of hartshorn or other alkali; at other times by applying a poultice made of common table-salt and the yolk of an egg.

Burns and Scalds are instantly relieved by immersing the parts in cold water; then send for the doctor, and while he is coming, cover the injured parts half an inch deep with common dry flour; keep the bowels acting daily, and take nothing but gruel, soups, stale bread, and baked fruits. This is the safest, best, speediest, and most certain cure ever made known for burns.

Colds.—Swallow not a morsel, cover up warm in bed, and remain there until well, drinking most freely of warm teas of any kind. If these things are done the day a cold is taken, they will seldom fail of a cure within thirty-six hours.

Corns.—After bathing the feet twenty minutes in hot water, rub a few drops of sweet oil on the corn with the finger for two or three minutes. Do this every night, and protect the corn from the pressure of a tight shoe or the chafing of a loose one, and the corn can be easily picked out with the finger-nail in a few days, not to return for months, if ever, when renew the treatment.

Cough of Children.—Keep them warm in a warm room, eating nothing but broths, stale bread, gruels, and baked fruits, and every four or five hours rub into the skin all over the chest, with the hand, patiently, half a teaspoonful of common sweet oil; taking every few hours during the day, a teaspoonful at a time, of sweet cider boiled to a syrup, which will keep a year in a cool cellar.

Diarrhea of summer is often cured by maintaining perfect quietude on a bed, and eating acid fruits or berries in their natural state while ripe, raw, and fresh. If the acid fruit fails, eat nothing for a few days but common rice, parched brown like coffee, then prepare in the usual way.

Erysipelas.—Keep the parts covered well with a poultice made of raw cranberries pounded. This disease comes without warning, and often ends fatally in a few days.

Odors—Of places, keep then clean; of persons, arising from a scrofulous taint, wash face, hands, neck, arms, arm-pits, and feet daily in a basin of water, in which has been mixed two table-spoonfuls of the compound tincture of spirits of hartshorn.

Poison.—Whether animal, vegetable or mineral, if swallowed, will be instantly neutralized in most cases by drinking a teacupful of common sweet oil promptly. The Choctaw Indians are said to rely on this as an infallible cure for the bite of rattlesnakes; if Laudanum, drink every ten minutes or oftener, until the drowsiness goes off, strong coffee, each cup cleared with the white of one egg.

New Shoes made easy.—Before having your measure taken, put on two pair of thick woolen stockings, but don't tell Crispin.

Surfeit.—Careful and wise persons sometimes over-eat, and are foundered like a horse; walk in the open air (until freely relieved) with sufficient activity to keep up a moderate perspiration, then go to a warm room and remain with all the clothing on until cooled off, and take nothing for the next two meals but stale bread and some warm drink, so as to rest the stomach.

Teeth.—If the tooth aches, out with it and be done with it. From five years old up to twenty, a conscientious dentist should be required to examine each particular tooth most minutely every three months, and once a year thereafter: use nothing for plugs but the purest gold. For personal comeliness, comfort, and health no money is more remunerative than that given to a good dentist.

Work by the day and not by the job when you want exercise. By thus working slowly, you are not exhausted before you know it, and by going to a very warm room the moment the work is over, cooling off very slowly, a cold is avoided, as well as that soreness and stiffness of limbs next day, which is the result of fitful, hasty, and too violent exercise.

